# Cardamonin inhibits breast cancer growth by repressing HIF-1α-dependent metabolic reprogramming

**DOI:** 10.1186/s13046-019-1351-4

**Published:** 2019-08-27

**Authors:** Jinmei Jin, Shuiping Qiu, Ping Wang, Xiaohui Liang, Fei Huang, Hui Wu, Beibei Zhang, Weidong Zhang, Xinhui Tian, Ren Xu, Hailian Shi, Xiaojun Wu

**Affiliations:** 1 Shanghai Key Laboratory of Compound Chinese Medicines, Institute of Chinese Materia Medica, Shanghai University of Traditional Chinese Medicine, 1200 Cailun Road, Zhangjiang Hi-tech Park, Shanghai, 201203 China; 2 Markey Cancer Center, Department of Pharmacology and Nutritional Sciences, University of Kentucky College of Medicine, Biopharm 553, 789 S. Limestone, Lexington, KY 40536 USA; 3 Institute of Interdisciplinary Integrative Medicine Sciences, Shanghai University of Traditional Chinese Medicine, Shanghai, 201203 China

**Keywords:** Cardamonin, Breast cancer, Mitochondrial oxidative phosphorylation, Reactive oxygen species, Apoptosis, Cell metabolism

## Abstract

**Background:**

Cardamonin, a chalcone isolated from *Alpiniae katsumadai*, has anti-inflammatory and anti-tumor activities. However, the molecular mechanism by which cardamonin inhibits breast cancer progression largely remains to be determined.

**Methods:**

CCK-8 and Hoechst 33258 staining were used to detect cell growth and apoptosis, respectively. HIF-1α driven transcription was measured by luciferase reporter assay. Glucose uptake and lactate content were detected with 2-NBDG and L-Lactate Assay Kit. Cell metabolism assays were performed on Agilent’s Seahorse Bioscience XF96 Extracellular Flux Analyzer. Mitochondrial membrane potential was measured with JC-1 probe. DCFH-DA was used to measure ROS level. Protein expression was detected by western blotting assay. Immunohistochemistry was performed to measure the expression of HIF-1α, LDHA and CD31 in tumor tissues.

**Results:**

Cardamonin inhibited growth of the triple negative breast cancer cell line MDA-MB-231 in vitro and in vivo by suppressing HIF-1α mediated cell metabolism. Cardamonin inhibited the expression of HIF-1α at mRNA and protein levels by repressing the mTOR/p70S6K pathway, and subsequently enhanced mitochondrial oxidative phosphorylation and induced reactive oxygen species (ROS) accumulation. We also found that cardamonin inhibited the Nrf2-dependent ROS scavenging system which further increased intracellular ROS levels. Eventually, accumulation of the intracellular ROS induced apoptosis in breast cancer cells. In addition, cardamonin treatment reduced glucose uptake as well as lactic acid production and efflux, suggesting its function in repressing the glycolysis process.

**Conclusions:**

These results reveal novel function of cardamonin in modulating cancer cell metabolism and suppressing breast cancer progression, and suggest its potential for breast cancer treatment.

**Electronic supplementary material:**

The online version of this article (10.1186/s13046-019-1351-4) contains supplementary material, which is available to authorized users.

## Background

Breast cancer is the second leading cause of cancer death among women across the world [1]. Chemotherapy is a common therapeutic strategy for breast cancer patients. However, serious adverse effects such as digestive tract toxicity, cardiovascular system toxicity, skin toxicity, hepatotoxicity, and delayed wound healing compromise the clinical effects of current chemotherapy drugs [2]. Thus, development of new chemotherapy drugs with fewer side effects remains to be an urgent need for breast cancer treatment.

Electron leakage from mitochondrial electron transport is the main source of intracellular reactive oxygen species (ROS) [3, 4]. Although appropriate levels of ROS benefit cancer cell proliferation and growth, over-accumulation of ROS results in cell damage and apoptosis [3, 5]. Metabolic reprogramming is one of the hallmarks of cancer [6]. Different from normal cells to use mitochondrial oxidative phosphorylation (OXPHOS) for energy supply, most of breast cancer cells have increased aerobic glycolysis in both normal and hypoxia conditions, which produces energy and intermediate metabolites to support proliferation and growth [6–9]. Compared with mitochondrial OXPHOS, aerobic glycolysis generates less ROS, while promotes glutaminolysis and enhances pentose phosphate pathway (PPP) metabolism to facilitate the biosynthetic process [10, 11]. Therefore, targeting metabolic reprogramming of cancer cells may be a promising strategy for cancer treatment.

Hypoxia-inducible factor-1α (HIF-1α) is an important regulator of cell metabolism in various tumors [12]. Upon activation, HIF-1α enhances aerobic glycolysis by directly inducing expressions of many cell metabolism genes, including glucose transporters (GLUTs) such as GLUT1, 3, 4, pyruvate dehydrogenase kinase 1 (PDHK1) [13], lactate dehydrogenase A (LDHA) and monocarboxylate transporters (MCTs) such as MCT1 and MCT4 [12, 14–16]. It has been shown that PI3K/PTEN/AKT and RAS/RAF/MEK/ERK pathways participate in the regulation of HIF-1α expression. The PI3K/PTEN/AKT pathway enhances translation of HIF-1α in a mTOR dependent or independent manner [12, 17–19]. The RAS/RAF/MEK/ERK pathway induces HIF-1α expression at both translational and transcriptional levels [12, 20, 21].

Cardamonin is a chalcone isolated from *Alpinia katsumadai* with significant anti-tumor activities in lung cancer, colon cancer, nasopharyngeal carcinoma and glioblastoma [22–31]. Cardamonin also inhibits growth of chemotherapy-resistant breast cancer cells [26]. Although cardamonin has been identified as a Wnt and NF-κB inhibitor [22, 29, 32], the detailed molecular mechanism by which cardamonin inhibits breast tumor growth largely remains to be determined. In the present study, we showed that cardamonin significantly inhibited the growth of breast cancer in vivo and in vitro, which is most likely mediated by reprogramming cancer metabolism through inhibition of the HIF-1 pathway. These findings may facilitate the clinical application of cardamonin in breast cancer treatment.

## Materials and methods

### Cell culture

MDA-MB-231 cells were obtained from Cell Bank, Type Culture Collection of Chinese Academy of Sciences (Shanghai, China), and maintained in DMEM medium (Gibco, Cat. No.:11965–092) supplemented with 10% fetal bovine serum (FBS, Gibco, Cat. No.: 10099–141) and 1% penicillin & streptomycin (Meilunbio, Cat. No.:MA0110) in a humidified incubator containing 5% CO_2_ at 37 °C. MGC803 and HCT8 cells, also obtained from Cell Bank, Type Culture Collection of Chinese Academy of Sciences, were both cultured in RPMI 1640 medium (Meilunbio, Cat. No.: MA 0215). MCF7, gifted by Prof. Tu Hong from Shanghai Jiao Tong University (China), was maintained in DMEM medium supplemented with 10% FBS and 1% penicillin & streptomycin. MCF-10A cells and BT549 cells, obtained from Zhongqiao Xinzhou Biotechnology (Shanghai, China), were cultured in special medium (Cat. No.: ZQ-1311, Zhongqiao Xinzhou Biotechnology) and RPMI 1640 medium, respectively, supplemented with 10% FBS and 1% penicillin & streptomycin.

### Cell viability assay

Cells were seeded in 96-well culture plates (2.0 × 10^3^ cells/well) and grown overnight. After treatment with cardamonin at different concentrations for 24–72 h, the cells were incubated with CCK-8 (Cell Counting Kit-8, DOJINDO Laboratories, Cat. No.: CK04) solution (20 μl/well) and cultured at 37 °C for another 1 h. Absorbance of the dissolved solutions was detected at 450 nm on a Thermo Scientific Varioskan Flash microplate reader (USA). The cell viability rate was calculated as follows: (absorbance of drug-treated sample/absorbance of control sample) × 100.

### Hoechst 33258 staining

MDA-MB-231 cells were seeded at a density of 1.5 × 10^5^ cells/ml on coverslips in a 24-well plate and allowed to adhere to the coverslips overnight. After being treated with cardamonin (10, 20 and 40 μM) for 24 h, the cells were fixed with 4% PFA for 10 min. Then being gently rinsed with 1 × PBS, the cells were stained with 10 μg/ml Hoechst 33258 solution for 15 min. Finally the cells were rinsed with 1 × PBS and the cell morphology was observed under a fluorescence microscope.

### Western blotting assay

MDA-MB-231 cells and tumor tissue homogenates were lysed in CelLytic™ MT Cell Lysis Reagent (Sigma, Cat. No.:C3228) containing protease and phosphatase inhibitors (Roche, Cat. No.: 04693116001, 04906837001) on ice for 30 min. After centrifugation at 12000 rpm for 15 min at 4 °C, the supernatant was collected and subjected to BCA assay to determine the protein concentration. Totally 30 μg proteins from each samples were separated by SDS-PAGE (10%) and transferred onto PVDF membrane. Afterwards, the membranes were blocked with 0.5% BSA for 1 h and incubated with primary antibodies against GAPDH (CST, Cat. No.:5174S, 1:1000), HIF-1α (BD, Cat. No.: 81095, 1:1000), PDHK1 (CST, Cat. No.: 3820 T, 1:1000), LDHA (CST, Cat. No.: c28H7, 1:1000), LDHB (Abcam, Cat. No.: ab85319, 1:1000), p-PI3K (CST, Cat. No.: Y458, 1:1000), PI3K (CST, Cat. No.: 4257S, 1:1000) p-AKT(CST, Cat. No.: S473, 1:1000), AKT (Abcam, Cat. No.: ab32505, 1:1000), p-mTOR (Abcam, Cat. No.: ab109268, 1:1000), mTOR (Abcam, Cat. No.: ab32028, 1:1000), P70S6K (CST, Cat. No.: 2903, 1:1000), p-p70S6K (Abcam, Cat. No.: 9234S, 1:1000), Cleaved-caspase3 (CST, Cat. No.: 9664S, 1:1000), Bcl2 (CST, Cat. No.: 50E3, 1:1000), Bax (CST, Cat. No.: 2772S, 1:1000), Nrf2 (Santa Cruz, Cat. No.: sc-722, 1:1000), NQO1 (Santa Cruz, Cat. No.: sc-32,793, 1:1000), and HO-1 (Santa Cruz, Cat. No.: sc-136,960, 1:1000) overnight at 4 °C. After being washed with 1 × TBST, the membranes were incubated with respective secondary antibodies conjugated with horseradish peroxidase for 1 h at room temperature. The protein bands were visualized with Immobilon™ Western Chemiluminescent HRP Substrate (Millipore Corporation, Cat. No.: WBKLS0500), and the images were captured on the visualization instrument Tanon-5200 (Tanon, China).

### Real-time quantitative PCR

Total RNA from MDA-MB-231 cells were extracted by using TRIzol Reagent (Ambion, REF: 15596018). cDNA was synthesized with Revert Aid First Strand cDNA Synthesis Kit (Thermo, Cat. No.: K1622). Real-time quantitative PCR was performed by using SYBR reagent (VazymE, L/N 7E141I7, Cat. No.: Q111–02) on Quant Studio 6 Flex System (Life technologies, Cat. No.: 20170777). Quantification of target genes was determined by the 2^−ΔΔCt^ method. And the relative expression of individual genes was normalized to that of GAPDH in the same sample. The sequences for forward (F) and reverse (R) primers used were listed as follows: HIF-1α, F: 5′-AGCCGAGGAAGAACTATGA-3′, R: 5′ -TTTGATGGGTGAGGAATG-3′; PDHK1, F: 5′- GATGTGAATGGGCAGTTAGT-3′, R:5′-AGGAATAGTGGGTTAGGTGAG-3′; LDHA, F: 5′- TGGAGTGGAATGAATGTTG-3′, R: 5′- GATGTGTAGCCTTTGAGTTTG-3′; LDHB, F: 5′- GAAGGAGGAAGAAGCACA-3′, R: 5′- GCACAAGGACAAGTAGGG-3′;GAPDH, F, 5′- GCACCGTCAAGGCTGAGAAC-3′, R: 5′- TGGTGAAGACGCCAGTGGA-3.

### Mitochondrial membrane potential (MMP) measurement

MMP of MDA-MB-231 cells were measured by using fluorescent probe JC-1 (Santa Cruz, Cat. No.: sc-364,116). The cells treated with cardamonin (20 μM) for different time points were rinsed with HBSS (Gibco, Cat. No.: 14025–092) and incubated with JC-1 (10 μM) at 37 °C for 30 min. Afterwards, the cells were rinsed with HBSS once again. Fluorescent intensity of the JC-1 monomers and aggregates was detected under different conditions (Ex (λ) 485 nm, Em (λ) 530 nm for monomers; Ex (λ) 530 nm, Em (λ) 590 nm for aggragates) on a microplate reader (Varioskan Flash, Thermo Scientific, UAS). Fluroscent images of the cells were taken under a fluorescent microscope (IX81, Olympus, Japan).

### Glucose uptake assay

Glucose uptake ability of MDA-MB-231 cells was evaluated by using the fluorescent glucose 2-NBDG (Thermo Fisher Scientific, Cat. No.: N13195). MDA-MB-231 cells seeded in 96-well plates were cultured in DMEM medium without glucose or carbon sources. After being treated with cardamonin (20 μM) for 3 and 6 h, respectively, the cells were gently rinsed with HBSS and incubated with 100 μM 2-NBDG at 37 °C for 30 min. Consequently, the cells were rewashed with HBSS. Fluorescent intensity of the cells was detected on a microplate reader (Ex (λ) 465 nm; Em (λ) 540 nm). Meanwhile, after being treated with cardamonin (10, 20, 40, 80 μM) for 6 h, the glucose uptake of MDA-MB-231 cells was also measured according to the above method.

### Lactate content measurement

Lactate, one of the end-products of aerobic glycolysis, can change the tumor microenvironment and have an impact on cancer-associated cells [10]. Intracellular and extracellular lactate contents of MDA-MB-231 cells were determined by using the L-Lactate Assay Kit (Cayman, Bath: 0521418, USA). MDA-MB-231 cells were cultured in the DMEM basic medium supplemented with 10% FBS and treated with cardamonin (20 μM) for 24 h. Then, the cells were cultured in DMEM basic medium containing 0.5% FBS for another 4 h. The cell pellet and culture medium were collected for lactate content measurement according to the manufacturer’s instructions.

### ROS measurement

The ROS level was measured according to the method described previously [33] with some modifications. Intracellular ROS level in MDA-MB-231 cells was detected by 2′, 7′-dichlorodihydrofluorescein (DCFH), which is oxidized into fluorescent dichlorofluorescein (DCF) in the presence of ROS. MDA-MB-231 cells were cultured in the 96-well plates at a density of 1 × 10^5^cells/ml in DMEM medium containing 10% FBS. After being treated with cardamonin (20 μM) for 2, 4, and 6 h, respectively, the cells were gently washed with HBSS followed by the incubation with 20 μM DCFH at 37 °C for 30 min. The dye was then removed and replaced with fresh HBSS. Fluorescence of the cells was measured immediately on a microplate reader (Ex (λ) 485 nm; Em (λ) 535 nm). Meanwhile, after being treated with cardamonin (10, 20, 40 and 80 μM) for 6 h, intracellular ROS level in MDA-MB-231 cells was also measured according to the above method.

### Cell metabolism assays

The Mito Stress Test Kit (Agilent, Cat. No.: 103015–100) was used to measure the oxygen consumption rate (OCR). The Glycolytic Rate Assay Kit (Agilent, Cat. No: 103344–100) was used for measuring the glycolytic proton efflux rate (GlycoPER). The Agilent Seahorse XF Real-Time ATP Rate Assay Kit (Agilent, Cat. No.: 103592–100) was used to detect the ATP production rates of mitochondrial oxidative phosphorylation and glycolysis, respectively. Before metabolism measurement, the probe plate was hydrated with HPLC grade water in a CO_2_-free incubator. The assay phenol red-free solution containing 10 mM glucose, 2 mM glutamine, 1 mM pyruvate and 5 mM HEPES was kept in a 37 °C CO_2_-free incubator to maintain the pH value. Then the HPLC grade water in the hydration plate was replaced with calibration solution and kept in a 37 °C CO_2_-free incubator. MDA-MB-231 cells were seeded into XF96 cell culture microplates (Seahorse Bioscience) at the density of 5000 cells/well (for measurement of OCR and GlycoPER) or 7500 cells/well (for measurement of ATP production rates of oxidative phosphorylation and glycolysis), and allowed to adhere to plate overnight. Then the cells were incubated with cardamonin (20 μM) for 3, 6 and 12 h, and the cell culture medium was replaced with phenol red-free assay solution and placed in a 37 °C CO_2_-free incubator for 1 h. Finally, OCR, GlycoPER and ATP production rates of mitochondrial oxidative phosphorylation and glycolysis were determined and analyzed on the Agilent’s Seahorse Bioscience XF96 Extracellular Flux Analyzer (Agilent Technologies) according to the manufacturer’s instructions and protocols (Seahorse Bioscience, North Billerica, MA, USA).

For the detection of GlycoPER value, Rot/AA (inhibitors of mitochondrial electron transport chain) and 2-deoxy-D-glucose (2-DG, inhibitor of glycolysis) were added according to the manufacturer’s instructions and protocols (Seahorse Bioscience, North Billerica, MA, USA).

For the measurement of OCR value, oligomucin, FCCP, and Rot/AA, respectively, were added according to the manufacturer’s instructions and protocols (Seahorse Bioscience, North Billerica, MA, USA).

For the determination of ATP production rates of mitochondrial oxidative phosphorylation and glycolysis, oligomycin and a mix of rotenone and antimycin A were added according to the manufacturer’s instructions and protocols (Seahorse Bioscience, North Billerica, MA, USA).

### HIF-1α overexpression induced by CoCl_2_

According to previous studies [34, 35], in this study, briefly, HIF-1α accumulation was induced by 100 μM CoCl_2_ (Cobalt chloride, Sigma-Aldrich, Cat. No.: 60818) for 6 h in MDA- MB-231 cells.

### Transient transfection for overexpression of PDHK1

GV230-PDHK1 plasmids or GV230 plasmids were purchased from Shanghai Genechem (Shanghai, China). Briefly, MDA-MB-231 cells at 60–80% confluency, was transiently transfected with GV230-PDHK1 plasmids or GV230 plasmids using NEOFECT DNA transfection reagent (Neofect Beijing Biotech, China). Results of western blotting assay (Fig.5g) demonstrated that PDHK1 overexpression was successfully induced after 32 h of transfection in MDA-MB-231 cells. In further study, MDA-MB-231 cells transiently transfected with GV230-PDHK1 plasmids or GV230 plasmids for 24 h were treated with or without cardamonin (20 μM) for 24 h. Then the cells were subjected to CCK-8 assay.

### Luciferase reporter assay

Luciferase reporter construct containing three HRE was purchased from Addgene (26731). HRE-luciferase reporter plasmid and Renilla luciferace vector were transiently transfected into HEK293 cells. The cells were treated with cardamonin (20 and 40 μM) for 24 h. Then the cells were harvested and subjected to luciferase activity assay by using Dual-Luciferase ^R^ Reporter Assay system (Promega, Cat. No. E1910).

### Animal treatment

Female nude mice, 4-week-old, were purchased from Shanghai Slake Experimental Animal Co., Ltd. and kept under SPF level conditions. All animal experiments were carried out in accordance with the protocol approved by the Animal Ethics Committee in Shanghai University of Traditional Chinese Medicine (SHUTCM), which complies with international rules and policies for laboratory animal use and care as founded in the European Community guidelines (EEC Directive of 1986; 86/609/EEC). All animal experiments were approved by the institutional Ethics Committee of SHUTCM (SZY201704012).

After habituation for a week, the mice were inoculated subcutaneously with MDA-MB-231 cells (1.0 × 10^7^cells in 200 μL PBS per mouse). When the tumor grew to 50 mm^3^, the nude mice were randomly divided into three groups, namely control group, cardamonin group and 5-Fu group. The mice in control group and cardamonin group were intraperitoneally injected with physiological saline and cardamonin (3 mg/kg), respectively, once a day. But the mice in 5-Fu group were intraperitoneally injected with 5-Fu (50 mg/kg) twice a week. Body weight and tumor size of the nude mice were recorded every 3 days. The size of the tumor was calculated according to the formula: [length × (width)^2^]/2. Four weeks after the drug administration, the nude mice were sacrificed and the tumor tissues were isolated for further analysis.

### Hematoxylin and eosin (H&E) staining

H&E staining was conducted to assess the pathological change of tumor tissues according to the procedure described previously [36]. And the images were captured with microscope (Olympus, BX61VS).

### Immunohistochemistry (IHC) analysis

The IHC experiments were performed according to the specific operations described previously [37–41]. In brief, tumor tissue sections, 5 μm thick, were deparaffinized with xylen and rehydrated in gradient alcohol (anhydrous, 95, 75%). After being washed three times in PBS, the sections were incubated with 0.3% H_2_O_2_ for 10 min to block the endogenous peroxidase. Then the sections were placed in sodium citrate antigen retrieval solution for 10 min in microwave oven. Thereafter, they were incubated with 10% goat serum blocking solution for 30 min followed by the incubation with primary antibodies against HIF-1α, LDHA and CD31 overnight at 4 °C. Subsequently, the slices were incubated with secondary antibody conjugated with horseradish peroxidase for 1 h at room temperature. After DAB coloring reaction, the images were captured by Olympus VS120 Virtual Slide Scanner. Positive expressions of target proteins were displayed in brown color, and the cell nuclei were stained in blue color by hematoxylin. Five randomly selected fields were evaluated using a BH2 microscope (Olympus, Tokyo, Japan) equipped with a Nikon 4500 digital camera. The area and optical density (OD) of HIF-1α, LDHA and CD31 in each field were processed by the computer-aided automatic image analysis system (Qiu Wei, Shanghai, China). The IHC index was calculated as the average integral OD: [(positive area×OD)/total area].

### Statistical analysis

All data were presented as mean ± standard deviation. Differences among groups were analyzed by one-way ANOVA analysis of variance with Dunnett’s test or the Student’s *t*-test using GraphPad 5.0 software (La Jolla, CA, USA). The value of *P* < 0.05 was considered statistically significant.

## Results

### Cardamonin inhibits cell viability and promotes apoptosis of MDA-MB-231 cells

**Fig. 1:**
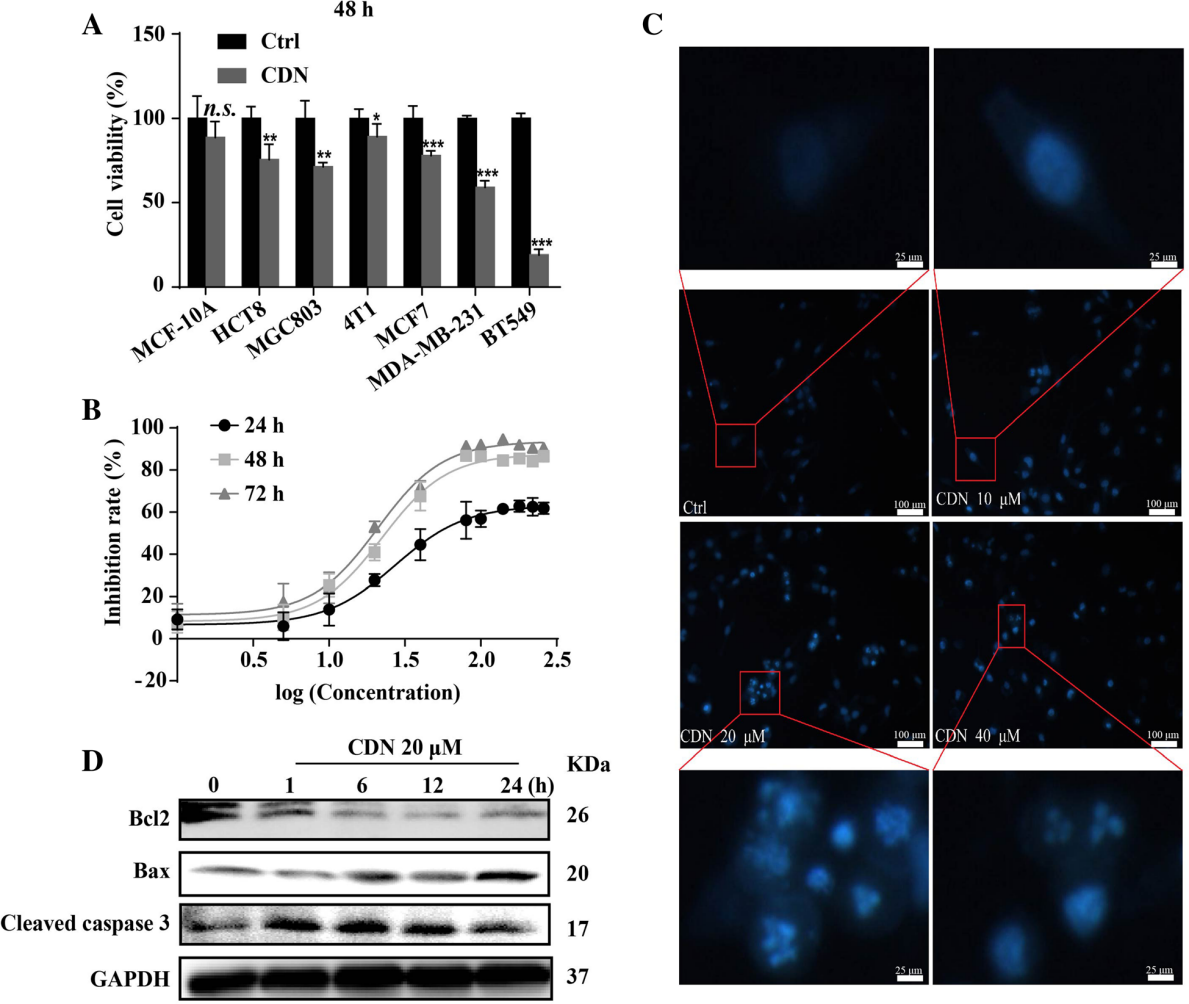
Cardamonin exhibits anti-cancer activity and induces cell apoptosis in vitro. **a** Quantification of inhibitory activity of cardamonin on cell viability in different cancer cell lines. The cell viability was determined by CCK-8 assays, after treatment with cardamonin (20 μM) for 48 h. **b** Cell viability of MDA-MB-231 cells was determined by CCK-8 assays after 24, 48 and 72 h of cardamonin treatment. **c** Cardamonin (20 and 40 μM) induced chromatin condensation and nuclear shrinkage or fragmentation in MDA-MB-231 cells after 24 h treatment; the nuclear structure was stained with Hoechst 33258. **d** Protein expression of apoptosis-related proteins in MDA-MB-231 cells treated with cardamonin (20 μM) for 1, 6, 12 and 24 h analyzed by Western blotting assay. Data are shown as mean ± SD; **, *P* < 0.01; ***, *P* < 0.001, compared with control group. *n* ≥ 3

To verify the inhibitory activity of cardamonin in tumor growth [22–31], we treated a panel of cancer lines with cardamonin in culture. Treatment with cardamonin (20 μM) for 48 h significantly inhibited cell viability of HCT8 (human colorectal cancer line) by 24.41%, MGC803 (human gastric cancer cell line) by 28.68%, MDA-MB-231 (human triple negative breast cancer (TNBC) cell line) by 40.93%, MCF-7 (human ER positive breast cancer cell line) by 21.83%, BT 549 (human TNBC cell line) by 85.70% and 4 T1 cells (mouse TNBC cell line) by 10.78%, but had little effect on cell viability of MCF-10A cells (human non-cancer mammary epithelial cell line) (Fig. 1a). As TNBC is an aggressive subtype of breast cancer with poor prognosis and has high metastasis and relapse rates after first-line treatment [42], we further investigated how cardamonin reduced viability of MDA-MB-231 cells. The IC_50_ values of cardamonin on MDA-MB-231 cells for 24 h, 48 h and 72 h, were 52.885 μM, 33.981 μM and 25.458 μM, respectively (Fig. 1b). We found that treatement with cardamonin (20 and 40 μM) induced chromatin condensation and nuclear shrinkage or fragmentation (Fig. 1c). Cardamonin (20 μM) also reduced protein levels of Bcl-2, while increased protein levels of Bax and cleaved caspase 3 in MDA-MB-231 cells (Fig. 1d). These results indicated that cardamonin treatment induced apoptosis in MDA-MB-231 cells.

Cardamonin also significantly inhibited cell viability of BT549 cells, and the IC_50_ values for the treatment of 24 h and 48 h, were 40.627 μM and 8.598 μM, respectively. The IC_50_ values of cardamonin on MCF7 cells for 24 h and 48 h, were 58.568 μM and 46.803 μM, respectively (Additional file 1: Figure S1).

### Cardamonin inhibits expression of HIF-1α and its target genes in MDA-MB-231 cells

**Fig. 2:**
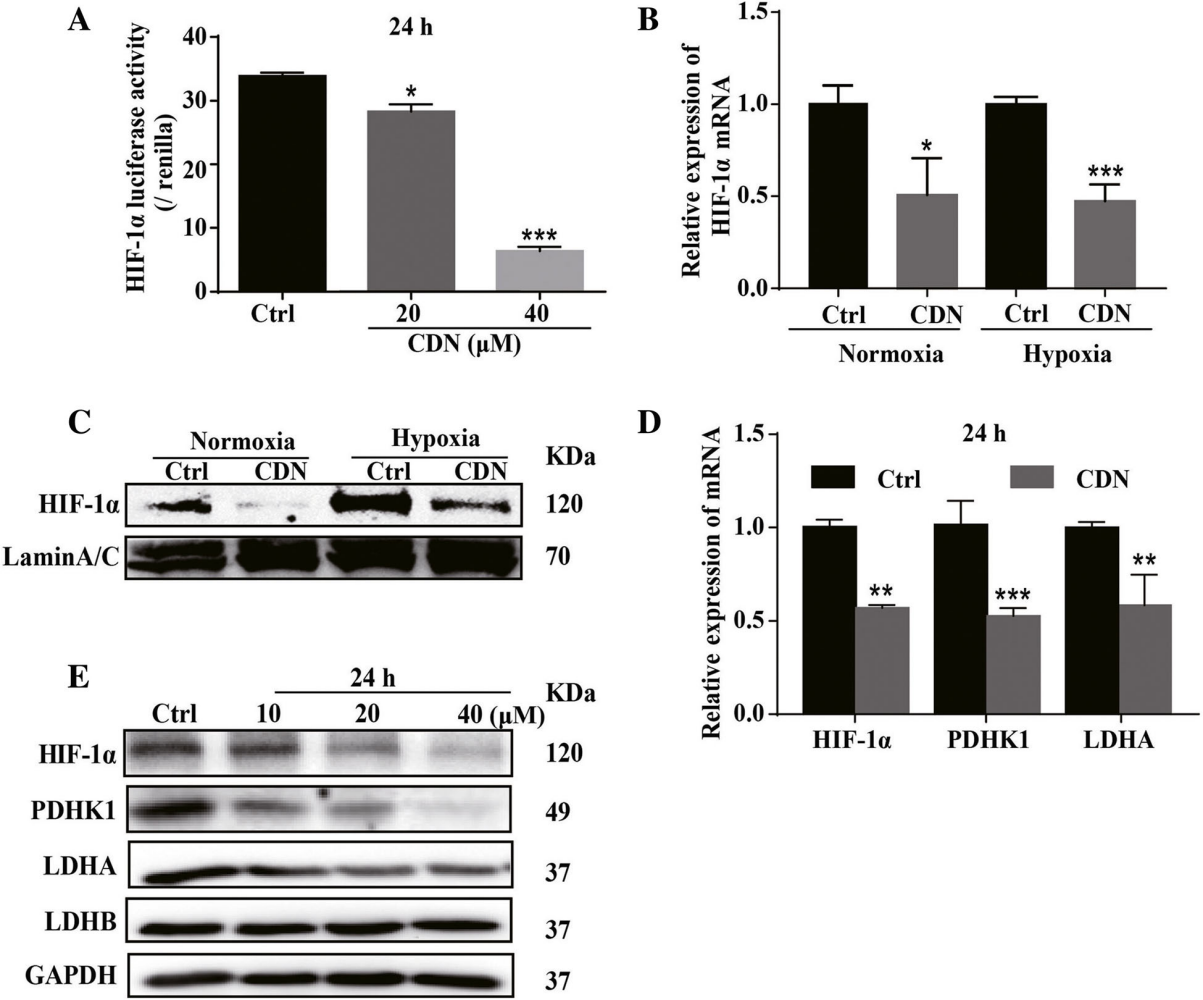
Cardamonin inhibits the HIF-1α pathway in MDA-MB-231 cells. **a** Luciferase report assay analyzed transactivational activity of HIF-1α in 293 T cells after 24 h treatment with cardamonin (20 and 40 μM). **b** qPCR analysis showed that treatment with cardamonin (20 μM) for 24 h reduced mRNA level of HIF-1α in MDA-MB-231 cells under both normal and hypoxia conditions. **c** Cardamonin (20 μM) treatment reduced the protein expression of HIF-1α in MDA-MB-231 cells under normal or hypoxic conditions. **d** qPCR results showed that treatment with cardamonin (20 μM) for 24 h reduced the mRNA levels of HIF-1α, PDHK1 and LDHA in MDA-MB-231 cells. **e** Treatment with cardamonin (10, 20 and 40 μM) for 24 h reduced the protein expression of HIF-1α, PDHK1 and LDHA in MDA-MB-231 cells. Data are shown as mean ± SD; *, *P* < 0.05, **, *P* < 0.01; ***, *P* < 0.001, ns means no statistical difference, compared with control group. *n* ≥ 3

HIF-1α over-expression is associated with advanced cancer progression and poor clinical outcomes in breast cancer patients [43, 44]. Activation of the HIF-1α pathway induces expression of many metabolism-related target genes such as PDHK1 and LDHA, which enhances glycolysis but reduces mitochondrial OXPHOS [42]. Using the HRE luciferease reporter construct that contains three hypoxia response elements (HRE), we showed that treatment with cardamonin significantly reduced HIF-1α driven transcription (Fig. 2a). Protein and mRNA levels of HIF-1α were downregulated in cardamonin treated cells under both normoxia and hypoxia conditions (Fig. 2b and c). Importantly, cardamonin (20 μM) treatment significantly inhibited transcription of HIF-1α target genes PDHK1 and LDHA (Fig. 2d). The protein levels of PDHK1 and LDHA were also reduced in cardamonin treated cells (Fig. 2e). Cardamonin treatment significantly inhibited the protein expressions of HIF-1α and PDHK1 in BT549 cells, but had no significant effect on protein levels of LDHA and LDHB (Additional file 1: Figure S3a-b). Interestingly, cardamonin treatment enhanced protein expression of HIF-1α, but reduced protein expression of PDHK1 in MCF7 cells (Additional file 1: Figure S3e-f), suggesting that other targets may mediate the inhibitory activity of cardamonin in MCF7 cells.

### Cardamonin induces metabolic reprogramming in breast cancer cell before apoptosis occurring

**Fig. 3:**
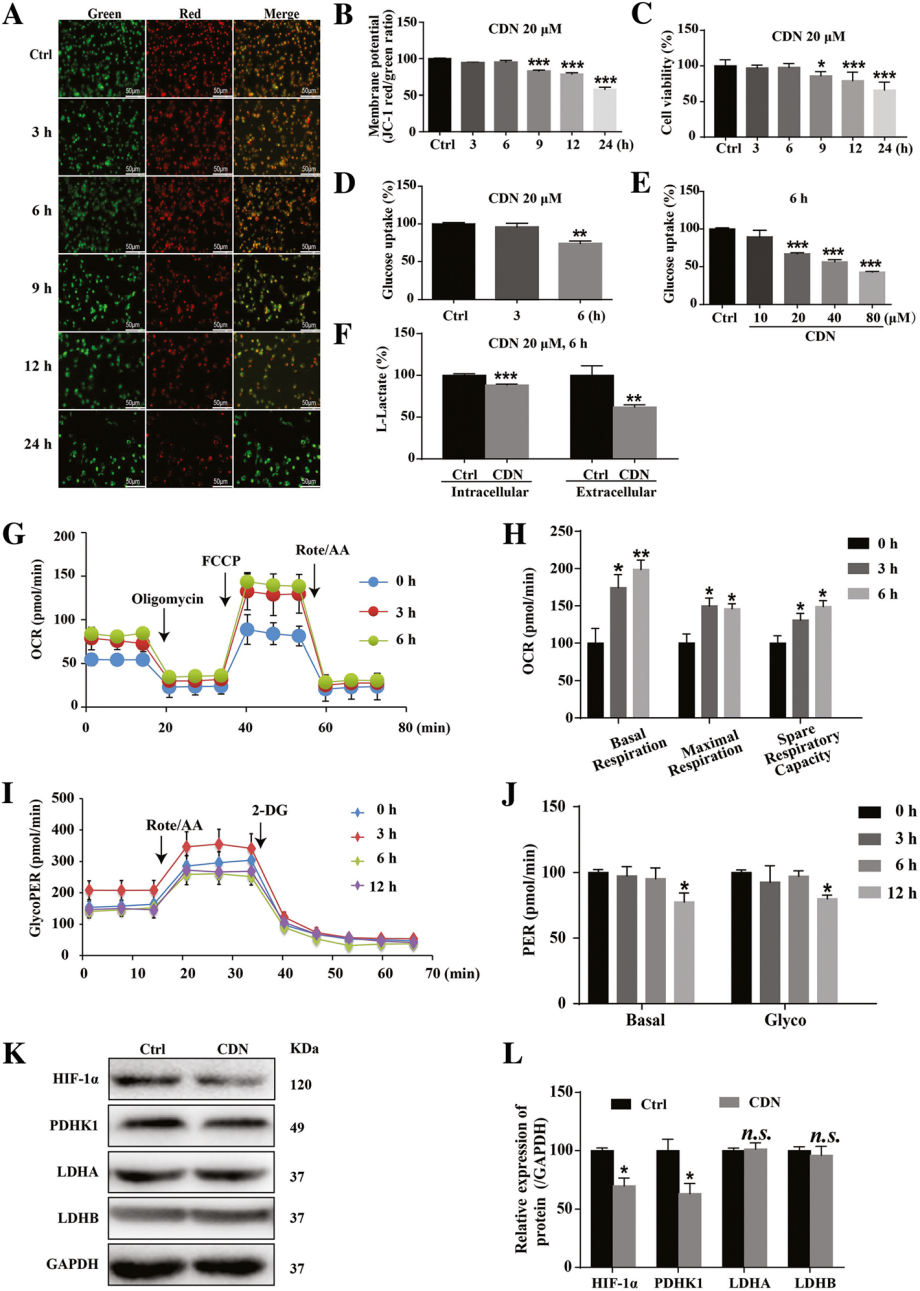
Cardamonin induces metabolic reprogramming in MDA-MB-231 cells before apoptosis occurring. **a**, **b** Cardamonin (20 μM) treatment decreased mitochondrial membrane potential (MMP) in MDA-MB-231 cells; the reduction of MMP was detected by JC-1 (10 μM) at 9 h after treatment. **c** CCK analysis showed that cardamonin (20 μM) inhibited cell viability in MDA-MB-231 cells at 9 h after treatment. **d**, **e** Treatment with cardamonin (20, 40 and 80 μM) for 6 h decreased glucose uptake in MDA-MB-231 cells in a dose-dependent manner. **f** Cardamonin (20 μM) treatment for 6 h decreased intracellular and extracellular lactate levels of MDA-MB-231 cells. **g**, **h** Treatment with cardamonin (20 μM) for 3 and 6 h significantly enhanced OCR in MDA-MB-231 cells. OCR and GlycoPER were detected by the Agilent’s Seahorse Bioscience XF96 Extracellular Flux Analyzer. **i**, **j** Cardamonin (20 μM) treatment for 12 h reduced glyco-PER of MDA-MB-231 cells. **k**, **l** Treatment with cardamonin (20 μM) for 6 h significantly reduced protein expression of HIF-1α and its metabolism related target genes. Data are shown as mean ± SD; *, *P* < 0.05, **, *P* < 0.01; ***, *P* < 0.001, compared with control. *n* ≥ 3. Scale bar, 50 μm

To determine function of cardamonin in cell metabolism, we first used JC-1 to examine the mitochondrial membrane potential in MDA-MB-231 cells. A reduction of the mitochondrial membrane potential was detected after cardamonin (20 μM) treatment for 9 h (Fig. 3a and b). Consistent with the decreased mitochondrial membrane potential, the cell viability was significantly decreased as early as 9 h after treatment (Fig. 3c). Next, we used fluorescent glucose 2-NBDG to measure glucose uptake in MDA-MB-231 cells. After 6 h treatment with cardamonin, glucose uptake was significantly decreased in MDA-MB-231 cells (Fig. 3d and e). Intracellular and extracellular L-lactate concentrations were also significantly decreased (Fig. 3f), indicating that cardamonin reduced L-lactate production and efflux. Results from the oxygen consumption rate (OCR) and extracellular acidification rate (ECAR) demonstrated that cardamonin (20 μM) greatly enhanced mitochondrial OXPHOS, as evidenced by increased basal respiration, maximal respiration and spare respiratory capacity, as early as 3 h after treatment (Fig. 3g and h). However, the basal PER and glyco-PER were significantly reduced at 12 h after cardamonin treatment (Fig. 3i and j), suggesting that the decrease of glycolytic rate was after the increase of mitochondrial OXPHOS. Interestingly, protein levels of HIF-1α and PDHK1 but not LDHA and LDHB, were significantly down-regulated at 6 h after cardamonin treatment (Fig. 3k-l). Same treatment also increased the oxygen consumption rate (OCR) in BT549 cells (Additional file 1: Figure S2a-b) and MCF7 cells (Additional file 1: Figure S2h-i). The basal PER and glyco-PER were significantly reduced in BT549 cells (Additional file 1: Figure S2c-d) and MCF7 cells (Additional file 1: Figure S2j-k) at 6 h after cardamonin treatment.

### Cardamonin treatment elevates ROS levels in MDA-MB-231 cells

**Fig. 4:**
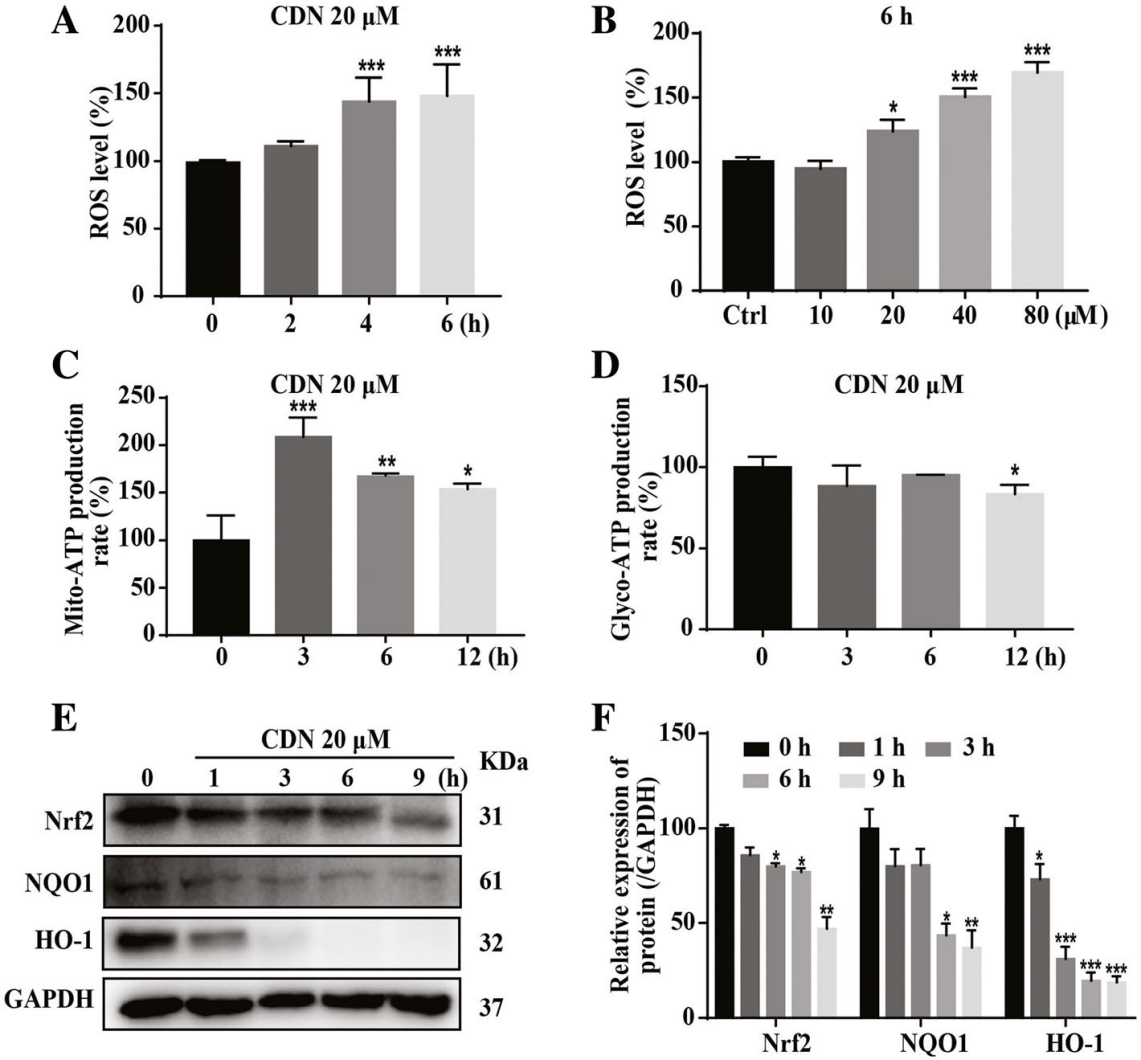
Cardamonin enhances ROS accumulation and reduces protein expression of Nrf2, NQO1 and HO-1. **a** DCFH-DA analysis showed that treatment with cardamonin (20 μM) for 4 and 6 h increased ROS accumulation in MDA-MB-231 cells. **b** Treatment with cardamonin (20, 40 and 80 μM) for 6 h increased ROS accumulation in MDA-MB-231 cells. **c** Treatment with cardamonin for 3, 6 and 12 h (20 μM) increased ATP production of mitochondrial oxidative phosphorylation in MDA-MB-231 cells. The ATP production was detected by the Agilent’s Seahorse Bioscience XF96 Extracellular Flux Analyzer. **d** Treatment with cardamonin (20 μM) decreased ATP production of glycolysis in MDA-MB-231 cells at 12 h. **e**, **f** Treatment with cardamonin (20 μM) for 3, 6 and 9 h reduced the protein expression of Nrf2, NQO1 and HO-1 in MDA-MB-231 cells. Data are shown as mean ± SD; *, *P* < 0.05, **, *P* < 0.01; ***, *P* < 0.001, ns means no statistical difference, compared with control. *n* ≥ 4

Mitochondrial OXPHOS generates significant amount of ATP, and ROS is a by-products of this process. Cardamonin treatment (20 μM) for 4 h elevated ROS levels in a dose-dependent manner (Fig. 4a and b). We also found that ATP production from mitochondria was enhanced by cardamonin (20 μM) (Fig. 4c); however, ATP production from glycolysis was decreased as late as 12 h after cardamonin treatment (Fig. 4d).

Nrf2, a stress-response transcription factor, regulates expression of antioxidant defense genes such as HO-1 and NQO1 by binding to anti-oxidant response elements (AREs) in promoter regions in the stress condition [5, 45–47]. Thus, activation of the Nrf2 defense system mitigates ROS accumulation [48]. Therefore, we asked whether the ROS scavenging system contributes to the cardamonin-induced ROS accumulation. The protein levels of Nrf2, HO-1 and NQO-1 were significantly reduced after cardamonin treatment for 6 h (Fig. 4e and f), suggesting that Nrf2 antioxidant pathway is a potential target of cardamonin that contributes to the ROS accumulation. Similar results were also obtained in BT549 (Additional file 1: Figure S2e-g) and MCF7 cells (Additional file 1: Figure S2l-n).

### Activation of HIF-1α pathway abolishes effects of cardamonin on mitochondrial OXPHOS and cell viability in MDA-MB-231 cells

**Fig. 5:**
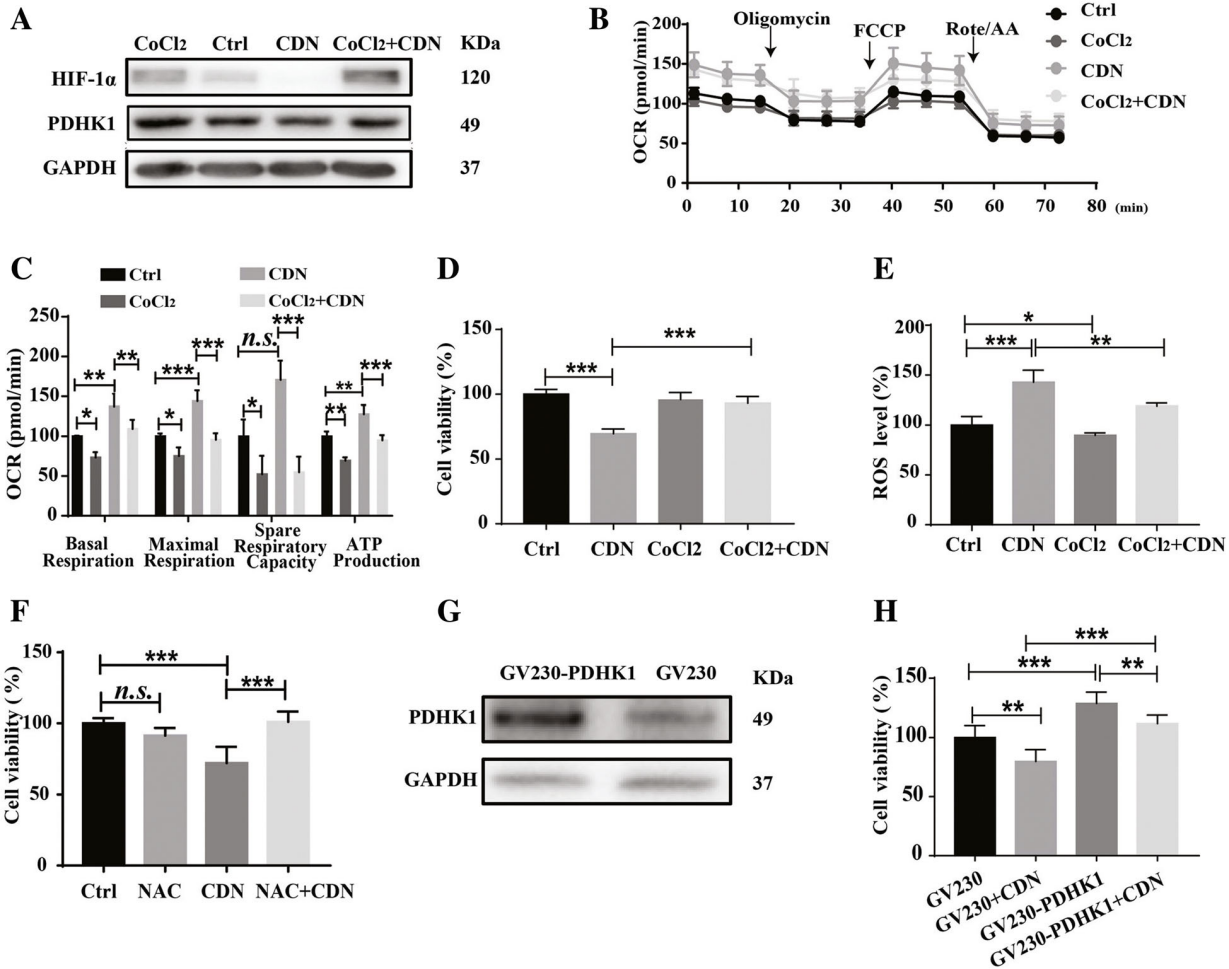
Restoring the HIF-1α/PDHK1 pathway abolishes the effect of cardamonin on mitochondrial oxidative phosphorylation and cell viability in MDA-MB-231 cells. **a** CoCl_2_ (100 μM) pre-stimulation for 6 h restored protein expression of HIF-1α and PDHK1 in cardamonin treated cells. **b**, **c** CoCl_2_ (100 μM) pre-treatment suppressed the cardamonin-induced OCR upregulation in MDA-MB-231 cells. **d** CoCl_2_ (100 μM) pre-treatment restored cell viability in cardamonin-treated MDA-MB-231 cells. **e** CoCl_2_ (100 μM) pre-treatment reversed the ROS accumulation in cardamonin-treated MDA-MB-231 cells. **f** NAC (N-acetyl-cysteine, ROS scavenger; 5 mM) pretreatment abolished the inhibitory effect of cardamonin (20 μM) on cell viability in MDA-MB-231 cells. **g** PDHK1 expression construct (GV230-PDHK1) was introduced in MDA-MB-231 cells by transiently transfection, and protein expression was assessed by western blot. **h** PDHK1 overexpression rescued cell viability in cardamonin-treated MDA-MB-231 cells. Data are shown as mean ± SD; *, *P* < 0.05, **, *P* < 0.01; ***, *P* < 0.001, ns means no statistical difference, compared with control. *n* ≥ 3

To determine whether the HIF-1α pathway mediated cardamonin’s function in regulating cell metabolism and viability, we performed rescue experiment by treating MDA-MB-231 cells with CoCl_2_. Treatment with CoCl_2_ (100 μM) increased protein levels of HIF-1α and induced PDHK1 expression in MDA-MB-231 cells. Importantly, CoCl_2_ treatment reversed the inhibitory effect of cardamonin on the protein expression of HIF-1α and PDHK1 (Fig. 5a). CoCl_2_ stimulation also counteracted the increased mitochondrial OXPHOS induced by cardamonin (Fig. 5b and c). Consistent with the above results, ROS production and accumulation were suppressed by CoCl_2_ in cardamonin-treated cells; the cell viability was also rescued (Fig. 5d and e). NAC (N-acetyl-cysteine, ROS scavenger; 5 mM) abolished the inhibitory effect of cardamonin on cell viability of MDA-MB-231 cells (Fig. 5f). To further confirm whether the downregulation of HIF-1α target genes mediated the inhibitory effect of cardamonin, we introduced the PDHK1 plasmid into MDA-MB-231 cells. We found that PDHK1 overexpression increased the cell viability of cardamonin-treated MDA-MB-231 cells (Fig. 5g and h). Similar results were also obtained in BT549 (Additional file 1: Figure S3c-d) and MCF7 cells (Additional file 1: Figure S3g-h).

### mTOR/p70S6K pathway is the target of cardamonin to inhibit the HIF-1α pathway

**Fig. 6:**
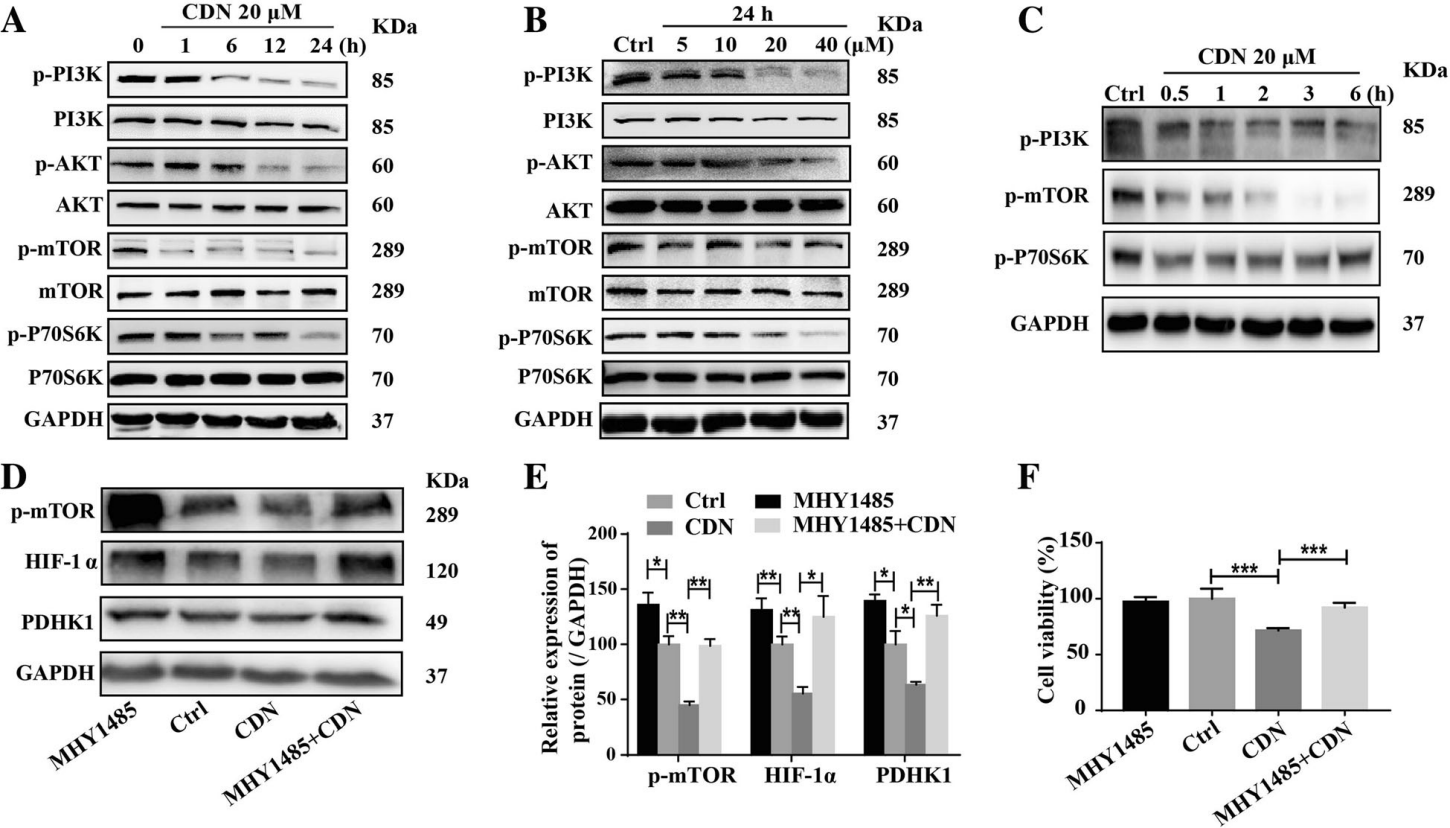
The mTOR/p70S6K pathway mediates the inhibitory effect of cardamonin on HIF-1α and cell viability. **a** Cardamonin (20 μM) treatment for 1, 6, 12 and 24 h reduced the phosphoryaltion of PI3K, AKT, mTOR and P70S6K in MDA-MB-231 cells. **b** Cardamonin (5, 10, 20 and 40 μM) treatment for 24 h decreased the phosphorylation of PI3K, AKT, mTOR and P70S6K in MDA-MB-231 cells. **c** Cardamonin (20 μM), as early after treatment for 0.5 h, mitigated the phosphorylation of mTOR and P70S6K in MDA-MB-231 cells. **d**, **e** Pretreatment with MHY1485 (mTOR activitor, 14 μM) for 30 min reversed the inhibitory effect of cardamonin (20 μM) on the protein expression of p-mTOR, HIF-1α and PDHK1. **f** CCK-8 analysis showed that pretreatment with MHY1485 rescued cell viability in cardamonin-treated MDA-MB-231 cells. Data are shown as mean ± SD; *, *P* < 0.05, **, *P* < 0.01; ***, *P* < 0.001. *n* ≥ 4

We found that cardamonin treatment (20 μM) reduced the protein expression of p-PI3K, p-Akt, p-mTOR and p-P70S6K in MDA-MB-231 cells in a time-dependent manner (Fig. 6a). Cardamonin treatment also decreased protein levels of p-PI3K, p-Akt, p-mTOR and p-P70S6K in a dose-dependent manner (Fig. 6b). However, at the early time points such as 0.5 h and 1 h, protein levels of p-mTOR and p-P70S6K but not p-PI3K, were significantly reduced in cardamonin-treated cells (Fig. 6c). These results suggested that the mTOR/P70S6K pathway may be upstream of the HIF-1α pathway in response to cardamonin treatment. Importantly, enhancing mTOR activity with MHY1485 rescued protein expression of HIF-1α and PDHK1 in cardamonin-treated MDA-MB-231 cells (Fig. 6d-e). Pretreatment with mTOR activator also attenuated the inhibitory effect of cardamonin on cell viability (Fig. 6f). These results indicate that cardamonin inhibited the HIF-1α pathway and cell viability through the mTOR/P70S6K pathway in MDA-MB-231 cells.

### Cardamonin inhibits tumor growth and protein expression of HIF-1α in the MDA-MB-231 xenograft model

**Fig. 7:**
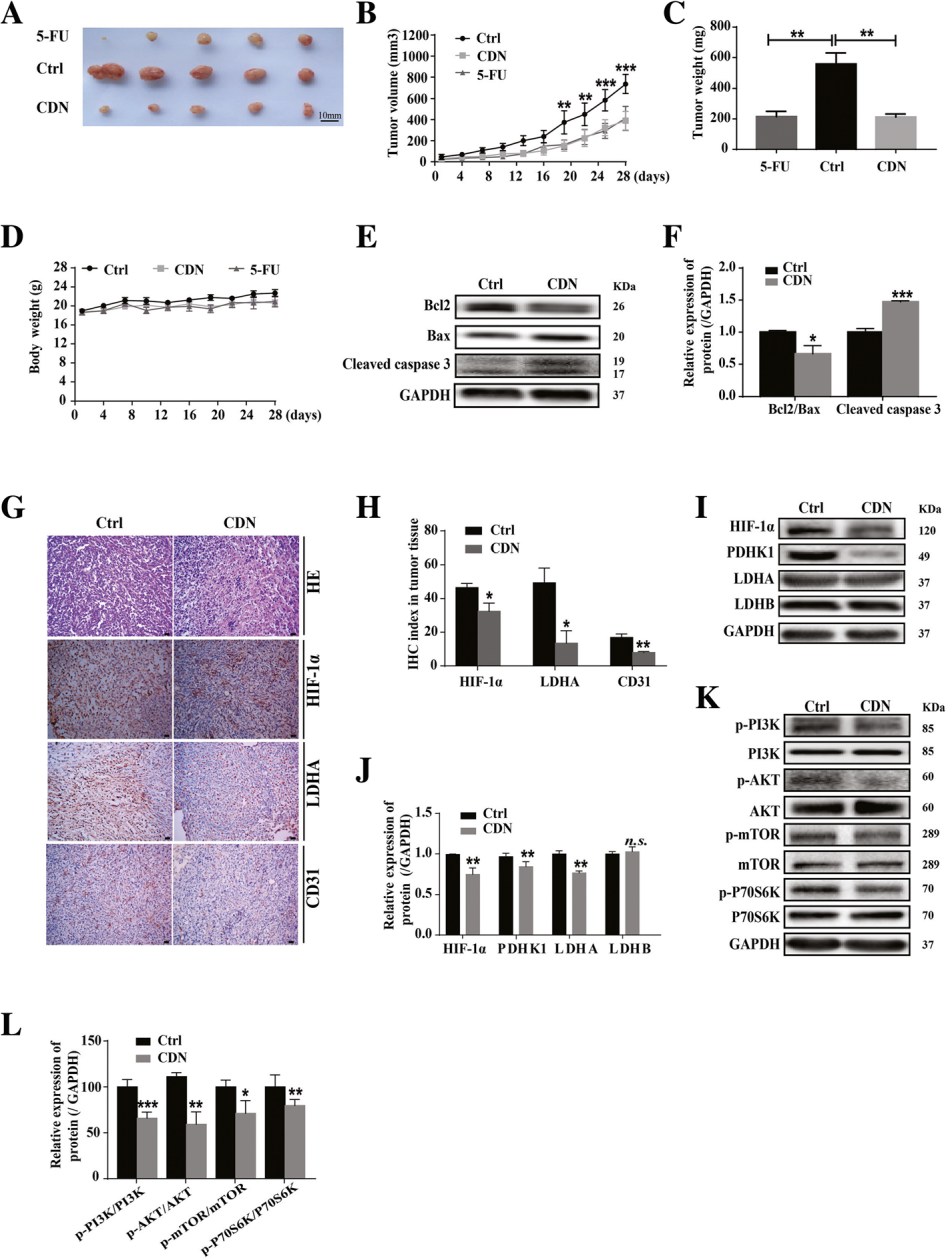
Cardamon inhibits tumor growth and the protein expression of HIF-1α and its metabolism- related target genes in the MDA-MB-231 xenograft model. **a** Cardamonin (3 mg/kg) significantly inhibited tumor growth. Scale bar, 10 mm. **b** Cardamonin (3 mg/kg) significantly inhibited tumors volume. The size of tumor was measured every three days and calculated according to the formula: [length × (width) ^2^]/2. **c** Cardamonin (3 mg/kg) treatment reduced the tumor weight. **d** Cardamonin (3 mg/kg) treatment had little effect on the body weight. **e**, **f** Cardamonin (3 mg/kg) reduced the ratio of Bcl2/Bax and enhanced protein expression of cleaved caspase 3 in tumor tissues. **g**, **h** Immunohistochemistry assay data showed that cardamonin (3 mg/kg) inhibited the protein expression of HIF-1α, LDHA and CD31 in tumor tissues (200×). **i**, **j** Western blotting analysis showed that cardamonin (3 mg/kg) inhibited the protein expression of HIF-1α, PDHK1 and LDHA in tumor tissues. **k**, **l** Cardamonin (3 mg/kg) inhibited the protein expression of p-PI3K, p-AKT, p-mTOR and p-p70S6K in tumor tissues. Data are shown as mean ± SD; *, *P* < 0.05, **, *P* < 0.01; ***, *P* < 0.001, ns means no statistical difference, compared with control. *n* ≥ 4

To verify the inhibitory effect of cardamonin on breast cancer growth in vivo, we performed drug treatment experiment with the MDA-MB-231 xenograft model. Cardamonin or 5-Fu administered by intraperitoneal injection significantly inhibited tumor growth in vivo compared with the control group (Fig. 7a, b and c). 5-Fu and cardamonin had little effect on the body weight of nude mice (Fig. 7d). Furthermore, the increased protein levels of cleaved caspase3 and Bax were upregulated in the cardamonin-treated group, while protein expression of Bcl2 and the ratio of Bcl2/Bax were reduced (Fig. 7e and f).

H&E staining results showed that tumor cells appeared necrotic and the cytoplasmic ratio of tumor cells were decreased after cardamonin treatment (Fig. 7g). To determine whether cardamonin inhibited the HIF-1α pathway in vivo, we assessed HIF-1α protein levels and its downstream targets in tumor tissues. IHC and western blot results showed that cardamonin treatment reduced protein levels of HIF-1α and LDHA in vivo (Fig. 7g-j). A reduction in protein levels of p-PI3K, p-AKT, p-mTOR, and p-P70S6K was detected in cardamonin-treated tumor tissues (Fig. 7k-l). We also found that CD31 staining reduced by 53.10% in cardamonin-treated breast cancer tissues (Fig. 7g and h), suggesting that cardamonin inhibits tumor angiogenesis.

## Discussion

In the present study, we identified novel function of cardamonin in regulating the HIF-1 pathway and cancer cell metabolism. We showed that cardamonin treatment reduced HIF-1α expression in TNBC cell lines, and subsequently inhibited glycolysis and increased mitochondrial OXPHOS and ROS accumulation, finally induced cell apoptosis in breast cancer cells in vitro and in vivo. These results suggest that cardamonin inhibits breast cancer progression by targeting the HIF-1 pathway and its mediated cancer cell metabolism.

ROS is mainly generated during mitochondrial OXPHOS [3, 4], and aerobic glycolysis may reduce ROS accumulation in tumor cells and enhance cancer cell survival during chemotherapy. Integrity of mitochondrial membrane is very important for mitochondrial OXPHOS; disruption of the membrane integrity not only impairs mitochondrial OXPHOS, but also induces cell apoptosis [49–53]. Treatment with cardamonin as late as for 9 h in breast cancer cells significantly reduced MMP, which was accompanied by a decrease in cell viability. And, enhanced mitochondrial OXPHOS and a reduction in glucose uptake, lactate production and lactate efflux were detected in cardamonin-treated MDA-MB-231 cells as early as for 6 h. These results suggest that cardamonin reduced cell viability by enhancing ROS generation in mitochondria.

Increased levels of ROS have been detected in many cancers, in which they promote tumor development and progression by inducing genome instability and multiple signaling pathways. However, accumulation of ROS to the high level also induces cell apoptosis, suggesting that a delicate balance of intracellular ROS levels is required for cancer cell function [53–55]. Most of the chemotherapeutic drugs and radiotherapy drugs induce cytotoxicity and cell cycle arrest by increasing oxidative stress [56–60]. We identified cardamonin as a ROS inducer in breast cancer cells. Cardamonin increased ROS at least partially through reducing the ROS scavenging system because Nrf2 and NQO1 expression is reduced upon treatment. Importantly, NAC, the active oxygen scavenger, reversed the inhibitory effect of cardamonin on cell viability in breast cancer cells. These results suggest that ROS is the critical mediator of inhibitory function of cardamonin in breast cancer cells.

Tumor recurrence and metastasis are major challenges in the treatment of tumors. Accumulation of lactate acid induced by glycolysis, which acidifies the tumor microenvironment and subsequently promotes tumor metastasis [60]. It has been shown that reduction of lactate acid level slowed down or even block cancer metastasis [60, 61]. We found that the lactate acid efflux was inhibited by cardamonin, implicating a potential function of cardamonin in suppressing cancer metastasis. Indeed, our unpublished data showed that cardamonin treatment inhibit breast cancer metastasis in vitro.

HIF-1α is a crucial regulator of cell metabolism by modulating glycolytic enzyme gene expression [12, 62, 63]. At the low level of HIF-1α, pyruvate, one of the end products of glycolysis, is mainly converted into acetyl-CoA and enters the citric acid cycle. However, the majority of pyruvate enters aerobic glycolysis when the HIF-1 pathway is activated. This regulation is mainly through PDHK1, a gene inhibiting PDH activity to reduce the entrance of acetyl-CoA into citric acid cycle [12, 62]. We found that cardamonin treatment decreased the mRNA and protein expressions of HIF-1α and inhibited its target genes in MDA-MB-231 and BT549 cells. Rescue experiments showed that introduction of HIF-1α and PDHK1 prevented the increase of mitochondrial OXPHOS and ROS accumulation induced by cardamonin. Together these results indicate that cardamonin inhibits cancer growth at least partially through targeting HIF-1α-mediated metabolic reprogramming in TNBC cells. In ER positive MCF7 cells, cardamonin failed to reduce protein levels of HIF-1α, but the protein expression of PDHK1 was still inhibited by cardamonin. However, PDHK1 overexpression or NAC both abolished the inhibitory effect of cardamonin on cell viability in MCF7 cells, suggesting that other molecular mechanisms are involved in PDHK1 regulation and metabolic reprogramming in MCF7 cells.

It has been shown that the mTOR/p70S6K pathway [19], NF-κB pathway [64, 65] and MAPK pathway [66, 67] regulate the HIF-1 pathway during cancer progression [68–70]. Our study showed that cardamonin inhibited activation of the mTOR/p70S6K pathway in MDA-MB-231 cells. Rescue experiments demonstrated that mTOR activation abolished the inhibitory activity of cardamonin on the cell viability and protein expression of HIF-1α and PDHK1 in MDA-MB-231 cells. Given roles of the NF-κB pathway in enhancing cell survival and HIF-1 activation, we also determined whether NF-κB mediates function of cardamonin in breast cancer cells. We found that cardamonin treatment reduced p-NF-κB levels at very early time point (Additional file 1: Figure S4). Interestingly, TNF-α (agonist of NF-κB) pretreatment reversed the inhibitory effect of cardamonin on cell viability and protein levels of p-NF-κB, HIF-1α and PDHK1 in MDA-MB-231 cells. This results suggest that the NF-κB pathway is another potential target of cardamonin that regulates HIF-1α/PDHK1 axis in breast cancer cells.

## Conclusions

**Fig. 8:**
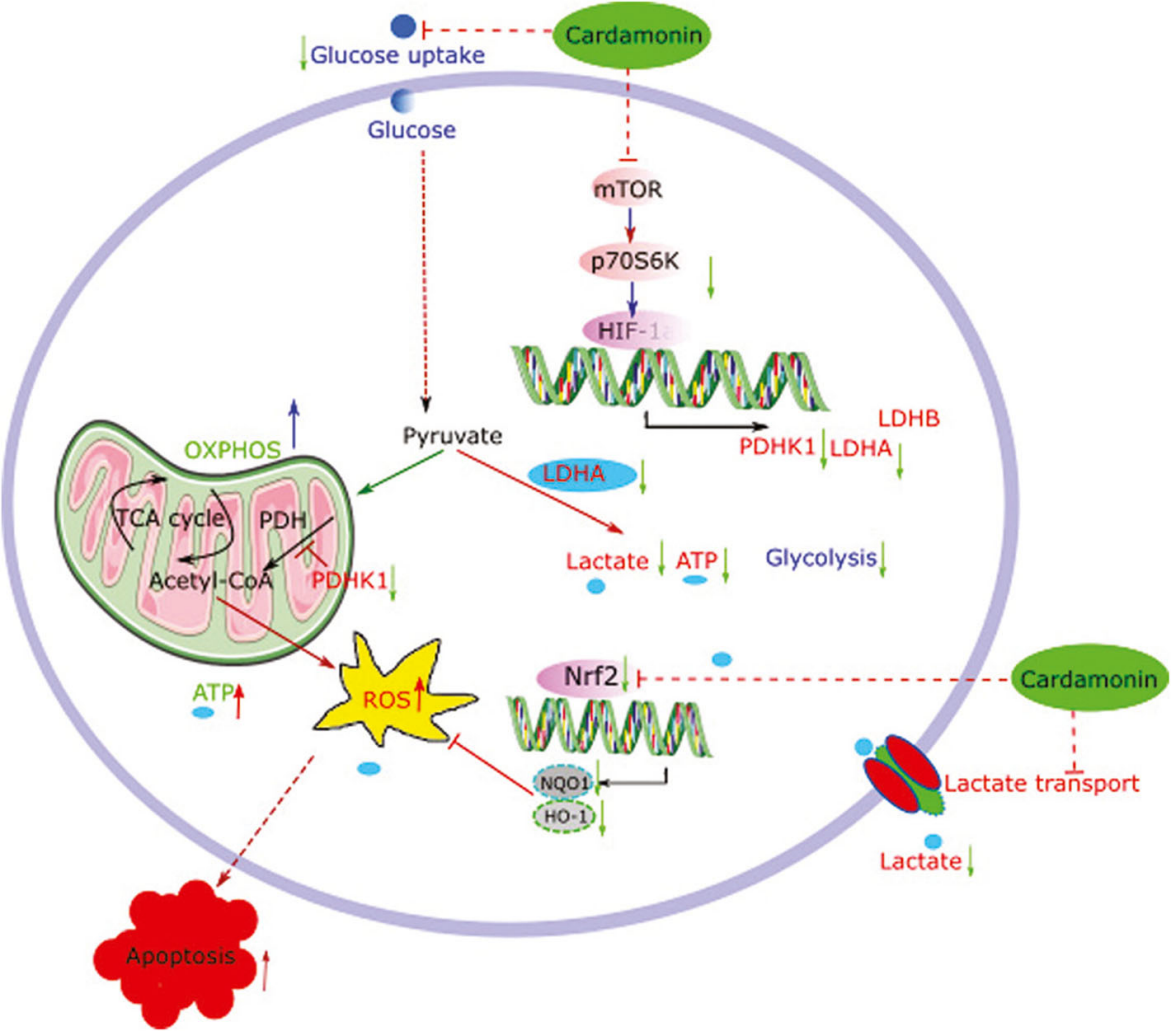
Schematic representation of possible molecular mechanism by which cardamonin represses HIF-1α-dependent metabolic reprogramming and inhibits breast cancer growth

Our results reveal novel function of cardamonin in inhibiting the HIF-1α pathway and its dependent metabolic reprogramming in breast cancer cells (Fig. 8). We also identified the mTOR/p70S6K pathway as one of the cardamonin targets to repress HIF-1α/PDHK1 axis. These findings provide the mechanistic insight into tumor inhibitory activity of cardamonin and suggest the therapeutic potential of cardamonin in breast cancer treatment.

## Additional file

Additional file 1 : **Figure S1.** Cardamonin inhibited cell viability of BT549 and MCF7 cells. **Figure S2.** Cardamonin regulated cancer metabolism and Nrf2 mediated antioxidant system in BT549 and MCF7 cells early before cell death. **Figure S3.** Cardamonin regulated HIF-1α/PDHK1 axis in BT549 and MCF7 cells. **Figure S4.** Cardamonin prevented the overactivation of NF-κB in MDA-MB-231 cells. (PDF 659 kb)

## Data Availability

All data that can prove the conclusion of this article are included in the article.

## Abbreviations

Akt: Protein kinase B, PKB
ATP: Adenosine triphosphate
CoCl_2_: Cobalt-chloride
Compensatory: Compensatory glycolysis
DCFH-DA: 2’,7’-Dichlorodihydrofluorescein
ECAR: Extracellular acidification rate
eIF4E: Eukaryotic initiation factor 4E
GLUTs: Glucose transporters
Glyco: Glycolysis
GlycoPER: Glycolytic proton efflux rate
HIF-1α: Hypoxia-inducible factor-1α
HO-1: Heme oxygenase-1
LDH: Lactate dehydrogenase
MCTs: Monocarboxylate transporters
MMP: Mitochondrial membrane potential
mTOR: Mammalian target of rapamycin
NAC: N-acetyl-L-cysteine
NQO1: NAD(P)H: quinone oxidoreductase 1
Nrf2: NF-E2-related factor 2
OCR: Oxygen consumption rate
OXPHOS: Mitochondrial oxidative phosphorylation
PDHK1: Pyruvate dehydrogenase kinase 1
PER: Proton efflux rate
PI3K: Phosphatidylinositol-3- kinase
PPP: Pentose phosphate pathway
ROS: Reactive oxygen species
