# Supplementary material for: Cardamonin inhibits breast cancer growth by repressing HIF-1α-dependent metabolic reprogramming
Source: J Exp Clin Cancer Res. 2019 Aug 27;38:377. doi: 10.1186/s13046-019-1351-4 (PMC6712736; doi:10.1186/s13046-019-1351-4)
Supplement: Supplementary file 1 — : Figure S1. Cardamonin inhibited cell viability of BT549 and MCF7 cells. Figure S2. Cardamonin regulated cancer metabolism and Nrf2 mediated antioxidant system in BT549 and MCF7 cells early before cell death. Figure S3. Cardamonin regulated HIF-1α/PDHK1 axis in BT549 and MCF7 cells. Figure S4. Cardamonin prevented the overactivation of NF-κB in MDA-MB-231 cells. (PDF 659 kb) [file 13046_2019_1351_MOESM1_ESM.pdf]

Supplementary materials and experiments

Cell culture.

MDA-MB-231 cells were obtained from Cell Bank, Type Culture Collection of Chinese Academy of Sciences (Shanghai, China), and maintained in DMEM medium (Gibco, Cat. No.:11965-092) supplemented with 10% fetal bovine serum (FBS, Gibco, Cat. No.: 10099-141) and 1% penicillin & streptomycin (Meilunbio, Cat. No.: MA0110) in a humidified incubator containing 5% CO_2 at 37 °C. MCF7 cells, gifted by Prof. Tu Hong from Shanghai Jiao Tong University (China), were maintained in DMEM medium (Gibco, Cat. No.:11965-092) supplemented with 10% FBS and 1% penicillin & streptomycin. BT549 cells, obtained from Zhongqiao Xinzhou Biotechnology (Shanghai, China), were cultured in RPMI 1640 medium, supplemented with 10% FBS (Gibco, Cat. No.: 10099-141) and 1% penicillin & streptomycin (Meilunbio, Cat. No.: MA0110).

Cell viability assay.

Cells were seeded at a density of 1.0×10^4 cells/ml (200 μl/well) in 96-well culture plates and grown overnight. After being incubated with cardamonin at different concentrations for different time points, the cells were incubated with CCK-8 (Cell Counting Kit-8, DOJINDO Laboratories, Cat. No.: CK04) solution (20 μl/well) and cultured at 37 °C for another 1 h. Absorbance of the dissolved solutions was detected at 450 nm on a Thermo Scientific Varioskan Flash microplate reader (USA). The cell viability rate was calculated as follows: (absorbance of drug-treated sample/absorbance of control sample) ×100.

Western blotting assay.

Breast cancer cells were lysed in CelLytic™ MT Cell Lysis Reagent (Sigma, Cat. No.:C3228) containing protease and phosphatase inhibitors (Roche, Cat. No.: 04693116001, 04906837001) on ice for 30 min. After centrifugation at 12 000 rpm for 15 min at 4°C, the supernatant was collected and subjected to BCA assay to determine the protein concentration. Totally 30 μg proteins from each samples were separated by SDS-PAGE (10%) and transferred onto PVDF membrane. Afterwards, the membranes were blocked with 0.5% BSA for 1 h and incubated with primary antibodies against GAPDH (CST, Cat. No.:5174S, 1:1000), HIF-1α (BD, Cat. No.: 81095, 1:1000), PDHK1 (CST, Cat. No.: 3820T, 1:1000), LDHA (CST, Cat. No.: c28H7, 1:1000), LDHB (Abcam, Cat. No.: ab85319, 1:1000), Nrf2 (Santa Cruz， Cat. No.: sc-722， 1:1000), NQO1 (Santa Cruz，Cat. No.: sc-32793，1:1000), HO-1 (Santa Cruz，Cat. No.: sc-136960， 1:1000), p-p65 NF-κB (CST， Cat. No.: 3033, 1:1000) overnight at 4 °C. After being washed with 1 × TBST, the membranes were incubated with respective secondary antibodies conjugated with horseradish peroxidase for 1 h at room temperature. The protein bands were visualized with Immobilon™ Western Chemiluminescent HRP Substrate (Millipore Corporation, Cat. No.: WBKLS0500), and the images were captured on the visualization instrument Tanon-5200 (Tanon, China).

ROS measurement.

Intracellular ROS level in BT549 and MCF7 cells was detected by 2’, 7’-dichlorodihydrofluorescein (DCFH), which is oxidized into fluorescent dichlorofluorescein (DCF) in the presence of ROS. BT549 and MCF7 cells were cultured in the 96-well plates at a density of 1×10^5/ml in DMEM medium containing 10% FBS. After being treated with cardamonin (20 μM) for 2, 4, and 6 h, respectively,

the cells were gently washed with HBSS followed by the incubation with 20 μM DCFH at 37°C for 30 min. The dye was then removed and replaced with fresh HBSS. Fluorescence of the cells was measured immediately on a microplate reader (Ex (λ) 485 nm; Em (λ) 535 nm). Meanwhile, after being treated with cardamonin (10, 20, 40 and 80 μM) for 6 h, intracellular ROS level in BT549 and MCF7 cells was also measured according to the above method.

Cell metabolism assays.

The Mito Stress Test Kit (Agilent, Cat. No.: 103015-100) was used to measure the oxygen consumption rate (OCR). The Glycolytic Rate Assay Kit (Agilent, Cat. No: 103344-100) was used for measuring the Glycolytic proton efflux rate (GlycoPER) . The Agilent Seahorse XF Real-Time ATP Rate Assay Kit (Agilent, Cat. No.: 103592-100) was used to detect the ATP production rates of mitochondrial oxidative phosphorylation and glycolysis, respectively. Before metabolism measurement, the probe plate was hydrated with HPLC grade water in a CO_2-free incubator. The assay phenol red-free solution containing 10 mM glucose, 2 mM glutamine, 1mM pyruvate and 5 mM HEPES was kept in a 37°C CO_2-free incubator to maintain the pH value. Then the HPLC grade water in the hydration plate was replaced with calibration solution and kept in a 37°C CO_2-free incubator. MDA-MB-231 cells were seeded into XF96 cell culture microplates (Seahorse Bioscience) at the density of 5000 cells/well (for measurement of OCR and GlycoPER) or 7500 cells/well (for measurement of ATP production rates of oxidative phosphorylation and glycolysis), and allowed to adhere to plate overnight. Then the cells were incubated with cardamonin (20 μM) for 3, 6 and 12 h, and the cell culture medium was replaced with phenol red-free assay solution and placed in a 37°C CO_2-free incubator for 1 h. Finally, OCR, GlycoPER

and ATP production rates of mitochondrial oxidative phosphorylation and glycolysis were determined and analyzed on the Agilent's Seahorse Bioscience XF96 Extracellular Flux Analyzer (Agilent Technologies) according to the manufacturer's instructions and protocols (Seahorse Bioscience, North Billerica, MA, USA).

For the detection of GlycoPER value, Rot/AA (inhibitors of mitochondrial electron transport chain) and 2-deoxy-D-glucose (2-DG, inhibitor of glycolysis) were added according to the manufacturer's instructions and protocols (Seahorse Bioscience, North Billerica, MA, USA).

For the measurement of OCR value, oligomucin, FCCP, and Rot/AA, respectively, were added according to the manufacturer's instructions and protocols (Seahorse Bioscience, North Billerica, MA, USA).

Transient transfection for overexpression of PDHK1.

GV230-PDHK1 plasmids or GV230 plasmids were purchased from Shanghai Genechem (Shanghai, China). Briefly, BT549 and MCF7 cells at 60-80% confluency were transiently transfected with GV230-PDHK1 plasmids or GV230 plasmids using NEOFECT DNA transfection reagent (Neofect Beijing Biotech, China). In further study, BT549 and MCF7 cells transiently transfected with GV230-PDHK1 plasmids or GV230 plasmids for 24 h were treated with or without cardamonin (20 μM) for 24 h. Then the cells were subjected to CCK-8 assay.

Figure legends

Figure S1 Cardamonin inhibited cell viability of BT549 and MCF7 cells. **a** Inhibitory activity of cardamonin at different concentrations on cell viability of BT549 cells. Cell viability was assessed after treatment with different concentrations of cardamonin (1-220 μM) for 24 h. **b** Inhibitory activity of cardamonin at different concentrations on cell viability of BT549 cells. Cell viability was assessed after treatment with different concentrations of cardamonin (1-220 μM) for 48 h. **c** Inhibition rates of cardamonin on BT549 cells after treatment for 24 and 48 h. **d** Cardamonin (5 μM) showed no significant inhibitory effect on cell viability of BT549 cells after treatment for 6 h. **e** Inhibitory activity of cardamonin at different concentrations on cell viability of MCF7 cells. Cell viability was assessed after treatment with different concentrations of cardamonin (1-220 μM) for 24 h. **f** Inhibitory activity of cardamonin at different concentrations on cell viability of MCF7 cells. Cell viability was assessed after treatment with different concentrations of cardamonin (1-220 μM) for 48 h. **g** Inhibition rates of cardamonin on MCF7 cells after treatment for 24 and 48 h. **h** Cardamonin (40 μM) treatment for 6 h showed no significant inhibitory effect on cell viability of MCF7 cells. Data are shown as mean ± SD; **, $P < 0.01$; ***, $P < 0.001$, compared with control group. n≥3.

Figure S2 Cardamonin regulated cancer metabolism and Nrf2 mediated antioxidant system in BT549 and MCF7 cells early before cell death. **a, b** Cardamonin (5 μM) significantly enhanced the OCR of BT549 cells. **c,d** Cardamonin (5 μM) reduced the PER and glyco-PER of BT549 cells. **e** Cardamonin (5 μM) significantly reduced the ROS accumulation in BT549 cells. **f, g** Cardamonin (5 μM) decreased Nrf2 and NQO1, but increased HO-1 in BT549 cells. **h, i** Cardamonin (40 μM) significantly

enhanced the OCR of MCF7 cells. **j, k** Cardamonin (40 μM) reduced the PER and glyco-PER of MCF7 cells. **l** Cardamonin (40 μM) significantly reduced the ROS accumulation in MCF7 cells. **m, n** Cardamonin (40 μM) reduced Nrf2 and NQO1, but increased HO-1 in MCF7 cells. All the measurements were carried out on cells treated with cardamonin for 6 h. Data are shown as mean ± SD; *, $P < 0.05$, **, $P < 0.01$; ***, $P < 0.001$, compared with control. n≥3.

Figure S3 Cardamonin regulated HIF-1α/PDHK1 axis in BT549 and MCF7 cells. **a**, **b** Cardamonin (5 μM) treatment for 6 h significantly reduced HIF-1α and PDHK1, but not LDHA and LDHB in BT549 cells. **c** Pretreatment with NAC (N-acetyl-cysteine, ROS scavenger; 5 mM) abolished the inhibitory effect of cardamonin (5 μM) on cell viability of BT549 cells. **d** Cardamonin (5 μM) treatment for 24 h did not inhibit the cell viability of BT549 cells overexpressed with PDHK1. **e**, **f** Cardamonin (40 μM) treatment for 6 h significantly increased HIF-1α and reduced PDHK1, but had no significant effect on LDHA and LDHB in MCF7 cells. **g** Pretreatment with NAC abolished the inhibitory effect of cardamonin (40 μM) on cell viability of MCF7 cells. **d** Cardamonin (40 μM) treatment for 24 h did not change the cell viability of MCF7 cells overexpressed with PDHK1. Data are shown as mean ± SD; *, $P < 0.05$, **, $P < 0.01$; ***, $P < 0.001$, ns means no statistical difference, compared with control group. n≥3.

Figure S4 Cardamonin prevented the overactivation of NF-κB in MDA-MB-231 cells. **a** Cardamonin (20 μM) treatment for 1, 3, and 6 h reduced the phosphorylated NF-κB in MDA-MB-231 cells. **b, c** Pretreatment with TNF-α (NF-κB activitor, 20 ng/ml) for 30 min reversed the inhibitory effect of cardamonin (20 μM) on the protein expression

of p-p65NF-κB, HIF-1α and PDHK1. **d** Pretreatment with TNF-α rescued cell viability in cardamonin-treated MDA-MB-231 cells. **e, f** Cardamonin (3 mg/kg) reduced the protein expression of p-p65NF-κB in tumor tissues

Figure S1

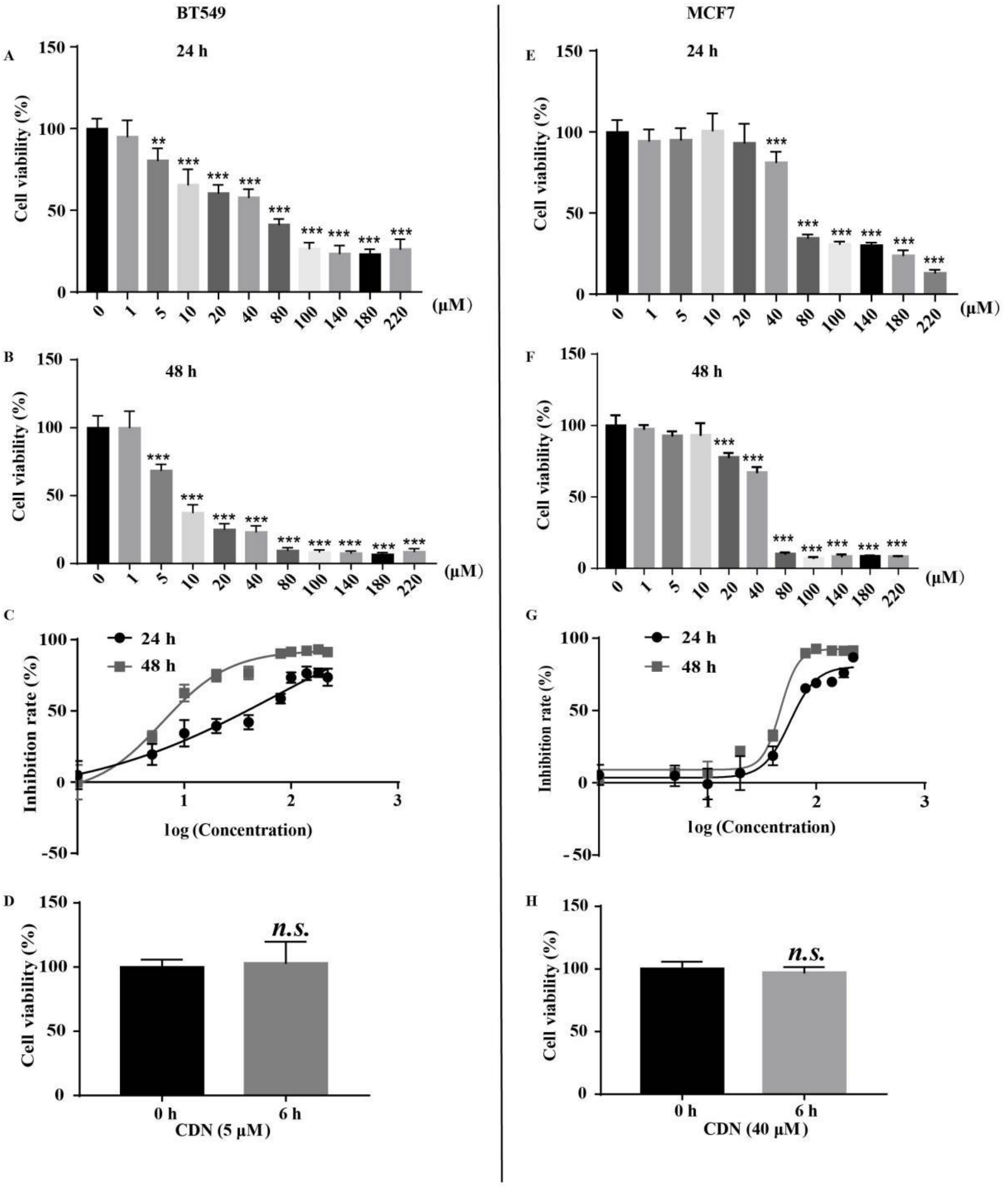
BT549
MCF7
A
B
C
D
E
F
G
H
24 h
48 h
Cell viability (%)
Inhibition rate (%)
log (Concentration)
(μM)
n.s.
0 h
6 h
CDN (5 μM)
CDN (40 μM)

Figure S2

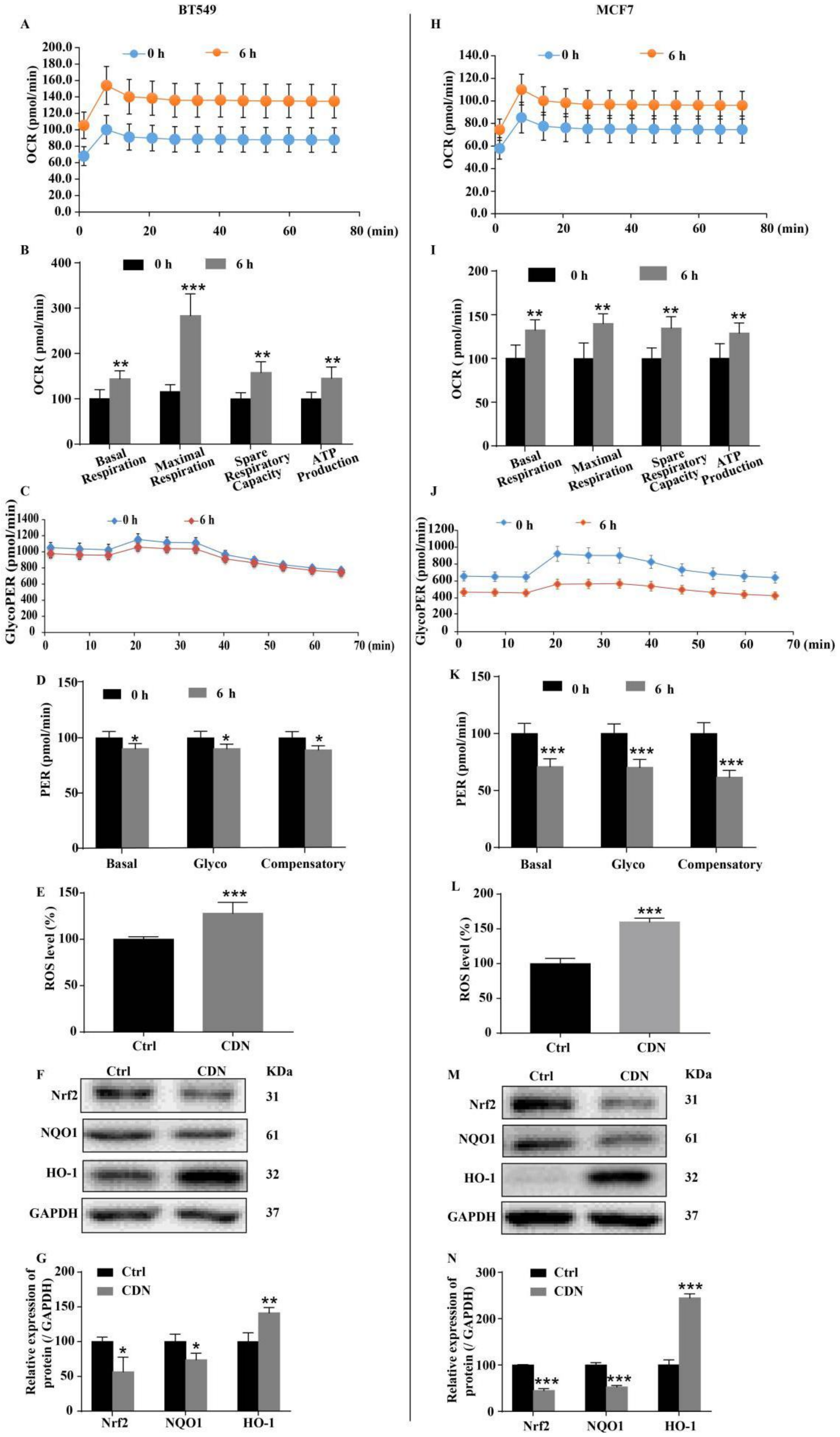
BT549
MCF7
A
B
C
D
E
F
G
H
I
J
K
L
M
N
0 h
6 h
OCR (pmol/min)
OCR (pmol/min)
80 (min)
70 (min)
Basal Respiration
Maximal Respiration
Spare Respiratory Capacity
ATP Production
GlycoPER (pmol/min)
PER (pmol/min)
Basal
Glyco
Compensatory
ROS level (%)
Ctrl
CDN
KDa
Nrf2
NQO1
HO-1
GAPDH
31
61
32
37
Relative expression of protein (/ GAPDH)

Figure S3

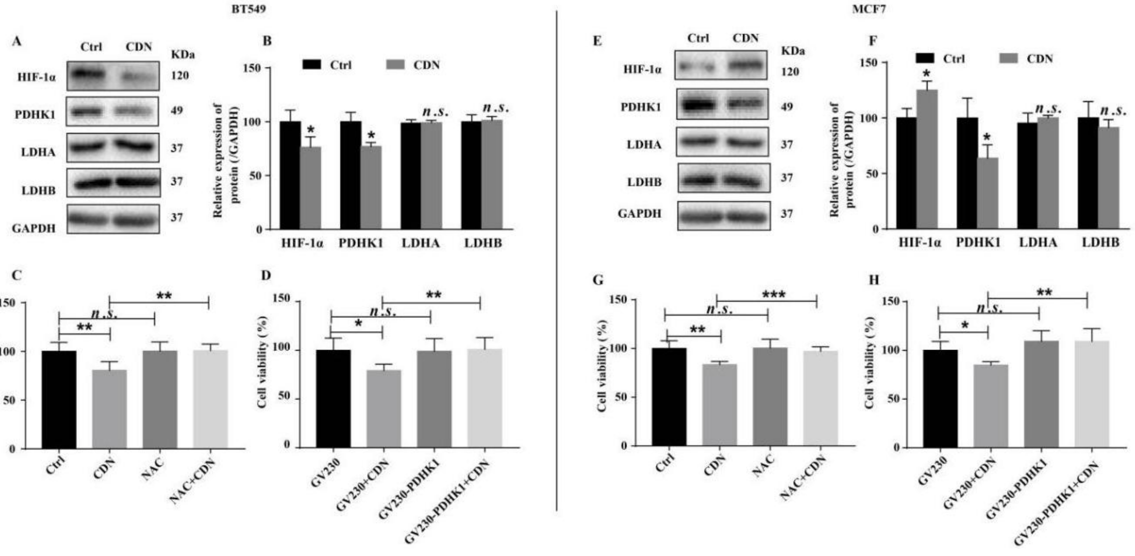
BT549
MCF7
A
Ctrl
CDN
KDa
HIF-1α
120
PDHK1
49
LDHA
37
LDHB
37
GAPDH
37
B
Ctrl
CDN
Relative expression of protein (/GAPDH)
150
100
50
0
HIF-1α
PDHK1
LDHA
LDHB
n.s.
C
Cell viability (%)
Ctrl
CDN
NAC
NAC+CDN
D
GV230
GV230+CDN
GV230-PDHK1
GV230-PDHK1+CDN
E
F
G
H

Figure S4

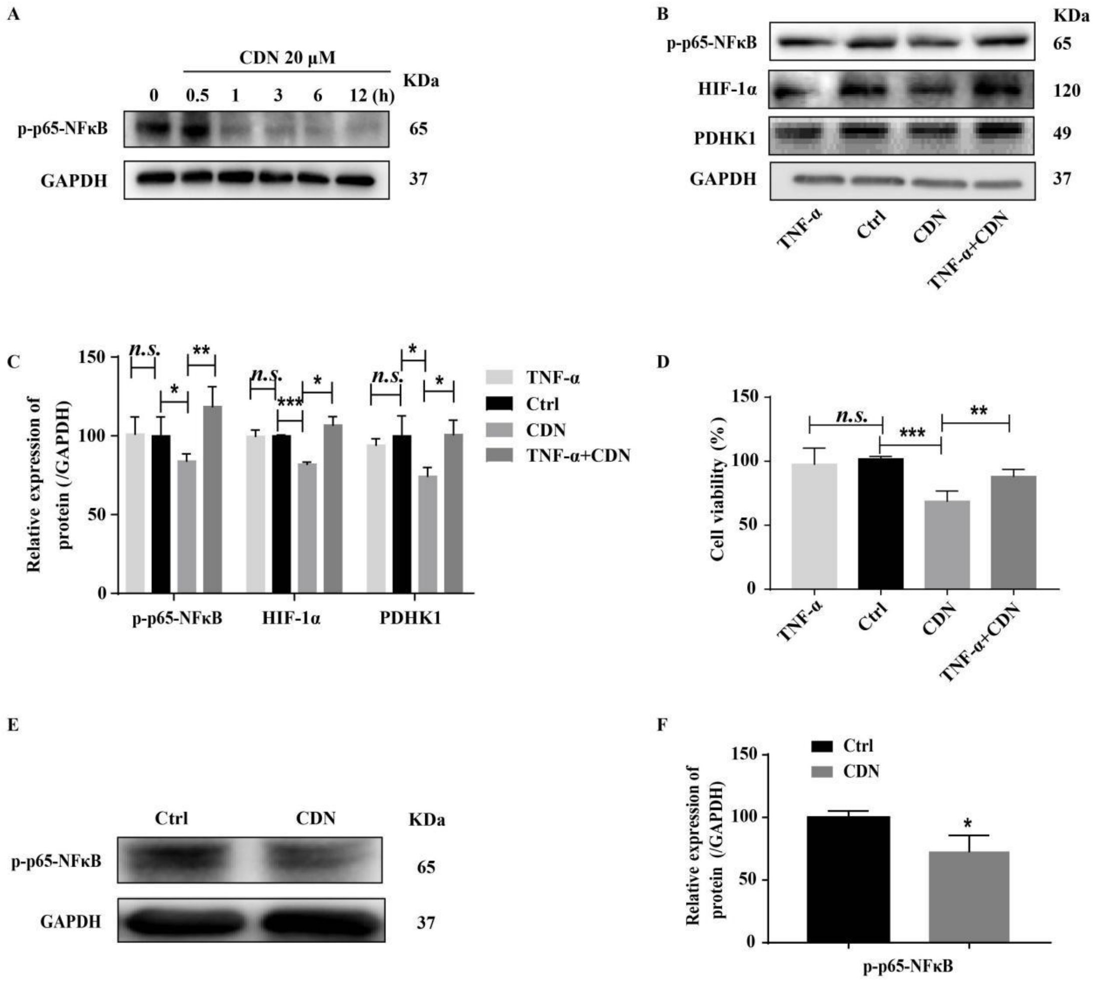

A
CDN 20 μM
0
0.5
1
3
6
12 (h)
KDa
p-p65-NFκB
65
GAPDH
37
B
KDa
p-p65-NFκB
65
HIF-1α
120
PDHK1
49
GAPDH
37
TNF-α
Ctrl
CDN
TNF-α+CDN
C
Relative expression of protein (/GAPDH)
150
100
50
0
n.s.
**
*
***
p-p65-NFκB
HIF-1α
PDHK1
TNF-α
Ctrl
CDN
TNF-α+CDN
D
Cell viability (%)
150
100
50
0
n.s.
***
**
TNF-α
Ctrl
CDN
TNF-α+CDN
E
Ctrl
CDN
KDa
p-p65-NFκB
65
GAPDH
37
F
Ctrl
CDN
Relative expression of protein (/GAPDH)
150
100
50
0
*
p-p65-NFκB
